# The Use of Direct Oral Anticoagulants (DOACs) in Older Adults Receiving Multidose Drug Dispensing; Interactions, Anticholinergic and Fall-Risk Increasing Drugs

**DOI:** 10.3390/geriatrics11020030

**Published:** 2026-03-06

**Authors:** Anette Vik Josendal, Ole Martin Sobakk, Anne Gerd Granas, Anne Katrine Eek

**Affiliations:** 1 Norwegian Centre for E-health Research, University Hospital of North Norway, 9038 Tromso, Norway; 2 Department of Pharmacy, The Faculty of Mathematics and Natural Sciences, University of Oslo, 0316 Oslo, Norway; ole_martins@hotmail.com (O.M.S.); anne.g.granas@uit.no (A.G.G.); 3 MedThings AS, 1624 Fredrikstad, Norway; 4 Department of Pharmacy, Faculty of Health Sciences, UiT—The Arctic University of Norway, 9019 Tromsø, Norway; 5 Regional Medicines Information and Pharmacovigilance Centre (RELIS), Oslo University Hospital, 0450 Oslo, Norway; anne.katrine.eek@ous-hf.no

**Keywords:** DOAC, multidose drug dispensing, fall-risk increasing drugs, anticholinergic drugs, drug–drug interactions, potentially inappropriate prescribing

## Abstract

**Objectives**: To examine the prescribing of non-vitamin K-dependent oral anticoagulants (DOACs) among multidose drug dispensing (MDD) users aged ≥65 years, and to describe associated drug–drug interactions (DDIs), concomitant use of fall-risk increasing drugs (FRIDs) and anticholinergic drugs (AC). **Methods:** Cross-sectional analysis of anonymized MDD medication lists from 87,519 patients in 2018. DDIs were identified using The Norwegian Medical Products Agency interaction tool, FRIDs were defined using the Swedish National Board of Health and Welfare list, and the CRIDECO Anticholinergic Load Scale assessed anticholinergic burden. **Results**: Among the 13,215 patients aged 65 and older the mean number of prescribed medications was 10.3. At least one DDI involving the prescribed DOACs was present in 26.8% of patients, whereas severe DDIs were rare (0.2%). Almost all (96.7%) used at least one FRID, and nearly half (46.8%) had an anticholinergic score ≥ 3. **Conclusions**: DOACs are frequently prescribed together with medications that increase the risk of falls and bleeding. These findings highlight the need for individualized risk–benefit evaluations and deprescribing or substituting high impact FRIDS and ACs when clinically appropriate.

## 1. Introduction

Stroke is the third-leading cause of death and disability combined and is estimated to cost US$721 billion worldwide [1]. One-third of ischaemic stroke patients have atrial fibrillation (AF) [2,3], a condition particularly common in older adults, as approximately 7–10% of people in their 70s and 15–18% of people in their 80s are diagnosed with AF [4]. The use of DOACs reduces the risk of stroke with two-thirds for patients by AF compared to placebo [5,6]. Until about a decade ago, warfarin was the most commonly used anticoagulation therapy, but non-vitamin K-dependent oral anticoagulants (DOACs) now outnumber warfarin [7,8].

DOACs are shown to be equally or more effective in preventing stroke compared to warfarin and carry a lower risk of intracranial bleeding [9,10,11]; however, there are still concerns about side effects, especially gastrointestinal bleeding. Although DOACs have fewer drug–drug interactions (DDIs) than warfarin [12,13], these can be harder to detect in clinical practice because DOAC use does not require INR monitoring [7,14]. Reduced routine monitoring may also delay detection of medication non-adherence and other drug-related problems, potentially increasing the risk of clots or bleeding.

Physicians sometimes withhold anticoagulation therapy due to concerns about fall-related bleeding, particularly in the very old [15,16]. Studies show that up to 51% of people over the age of 85 fall annually [17]. Falls may result in decreased mobility and thereby further increase fall risk. Medicines that increase the risk of falling, often abbreviated FRIDs (fall risk-increasing drugs), are commonly prescribed to older adults. A Swedish study showed that among people over 65 years of age admitted to the hospital with a hip fracture, 93% were taking at least one FRID [18]. Although fall risk alone is not considered a sufficient reason to withhold anticoagulation therapy, one should aim to minimize the risk of falls caused by medicines [19].

Another group of medications that might increase the fall risk is anticholinergic drugs (AC), as these have side effects such as confusion and dizziness [20]. It is debated whether ACs have an independent association with falls, or whether this is related to dosage, duration of treatment, or other non-anticholinergic effects of such drugs [21]. Nevertheless, studies have shown that patients with a high anticholinergic score have an increased risk of falling [22,23,24].

Adverse effects from FRIDs, AC and DDI often overlap in the same patients and may interact with nutritional problems to worsen frailty, falls and functional decline [25]. Recognizing these overlapping risks therefore requires a holistic geriatric assessment that integrates systematic medication review (including deprescribing where appropriate), targeted nutritional evaluation and routine collection of simple laboratory markers to identify malnutrition [26]. Such an integrated approach helps clinicians prioritize modifiable contributors to pharmacologic risk and tailor anticoagulation decisions to each patient’s overall vulnerability. Therefore, describing common DOAC co-prescribing patterns that increase pharmacologic risk may help prioritize targets for holistic geriatric assessment and medication review. This study examines DOAC prescribing in patients aged ≥65 years, focusing on co-prescribing patterns associated with increased pharmacologic risk (drug–drug interactions, fall-risk-increasing drugs and anticholinergic drugs).

## 2. Materials and Methods

### 2.1. Study Design and Sample

This is a cross-sectional study using anonymized multidose prescriptions from June 2018. The dataset was provided by an MDD supplier that dispensed nearly 90% of all MDDs in Norway at the time. The medication lists included drug names, ATC codes, strength of the medication, formulation, dosing schedule, dispensing type (regular medications or when-needed medication) and reimbursement codes. Patient data included age, gender and care setting (self-pay, home care service, or nursing home).

### 2.2. The Multidose Prescription

Multidose drug dispensing (MDD) is a dosing aid where tablets and capsules are machine-dispensed in disposable plastic bags. A multidose prescription lists all prescribed medications for a patient; in addition, it can include over-the-counter (OTC) medications and dietary supplements. The medication list has three parts: the multidose-dispensed drugs, other regular medications, and as-needed medications (prn.). The multidose-dispensed drugs are tablets and capsules with fixed dosing schedules. Other regular medications are those not suited for multidose bags, e.g., ointments or inhalers. In our study, medicines listed as MDD are dispensed medications, and medicines listed as other regular medications or as-needed medications represent prescribed medications but are not necessarily dispensed. When changes are made to an MDD patient’s prescribing to either of the three parts in the medication list, the GP transmits the complete medication list to the pharmacy. In this way, the pharmacy holds an up-to-date list of what the patient is actually prescribed at any time. The MDD prescription undergoes the standard prescription verification process in the pharmacy ordering the MDD and an approved MDD manufacturer performs the actual packaging. Detailed information about the MDD prescriptions and work processes in Norway is published elsewhere [27].

### 2.3. Outcome Measures

First, we calculated the use of warfarin and the four DOACs, dabigatran, rivaroxaban, apixaban and edoxaban, including indication for use. Then, each patient’s medication list was screened for DDI involving DOACs, concomitant use of DOACs and FRIDS, and of DOACs and ACs. Lastly, we calculated the anticholinergic burden for each patient.

Indication for the use of the DOAC was identified from the reimbursement code on prescriptions for patients in home care services and self-pay patients. Reimbursement codes were not available for prescriptions issued in nursing homes.

To identify DDIs, we combined two sources: the Summary of Product Characteristics (SPC) of the four DOACs published in The Norwegian Pharmaceutical Product Compendium [28], and DDIs from the European Society of Cardiology [29] and results were cross-checked against the ESC/EHRA practical guide as comparative reference for severity and recommended actions. ATC codes from the MDD medication lists were converted to active substance names and matched to SPC. The DDIs were classified as either “Type B: precautions should be taken” or “Type C: should be avoided”. To identify FRIDs, we used a list from the Swedish National Board of Health and Welfare [30]. To calculate an anticholinergic score, we used the CRIDECO Anticholinergic Load Scale (CALS) but excluded medications without market authorization in Norway [31]. CALS was chosen in favour of the more well-known Anticholinergic Drug Scale (ADS) and Anticholinergic Burden (ACB) scales [32] because CALS includes newer therapies which were not available at the time the other scales were developed. The CALS assigns each medication an anticholinergic score between 0 and 3. A patient’s anticholinergic burden is the sum of the scores for each medication, and a total score of 3 or more is considered clinically relevant [31].

### 2.4. Data Processing and Statistical Analysis

The original anonymized dataset was provided by the wholesaler Apotek 1 Gruppen AS, Lørenskog, Norway, who dispense MDD and comprised 87,519 patients and 859,642 medicines. We excluded 192 patients due to incomplete information on age or gender and 17,470 patients under the age of 65. After calculating the relative use of different types of anticoagulants, the remaining analyses were performed only on the 13,215 patients aged ≥65 years who were prescribed a DOAC. Analyses were performed in Stata 17.0. Means and standard deviations were used to describe sample characteristics. From age 65 onwards, age was grouped into 5-year intervals.

### 2.5. Ethics

This study was approved by the Data Protection Officer at Apotek 1 Gruppen AS Lørenskog, Norway. The data were anonymized by Apotek 1 before being given to the researchers, and the study did not require approval from the Regional Ethics Committee.

## 3. Results

**Figure 1:**
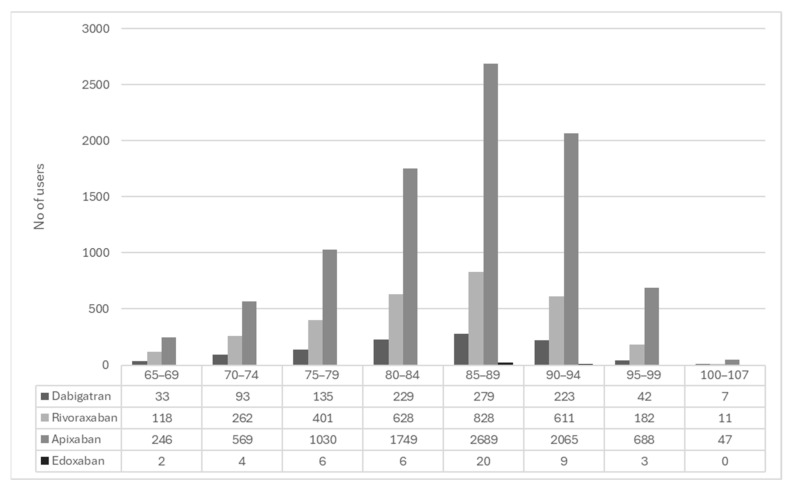
The use of the four DOACs in different age groups (*n* = 13,215).

The study population characteristics, including the relative use of the different types of DOAC, are shown in Table 1. Of the 87,519 MDD users in our dataset, 13,215 patients (18.9%) were prescribed a DOAC and 4137 (6.0%) were prescribed warfarin. The DOAC users had a mean age of 85; 38.5% were male and were on average prescribed 10.3 medications. Apixaban was the most used DOAC (68.7%) while edoxaban was the least used (0.4%). Figure 1 shows the age distribution of the study population and the relative use of the four DOACs.

In total, 84.0% had the indication for use listed on their prescription, where the two most common were atrial fibrillation and flutter (88.1%) and pulmonary embolism (5.8%).

Table 2 shows the prevalence of FRIDs, DDIs and ACs in this population, showing a higher prevalence of all three classes in patients in home care compared to nursing homes.

### 3.1. Drug–Drug Interactions

**Figure 2:**
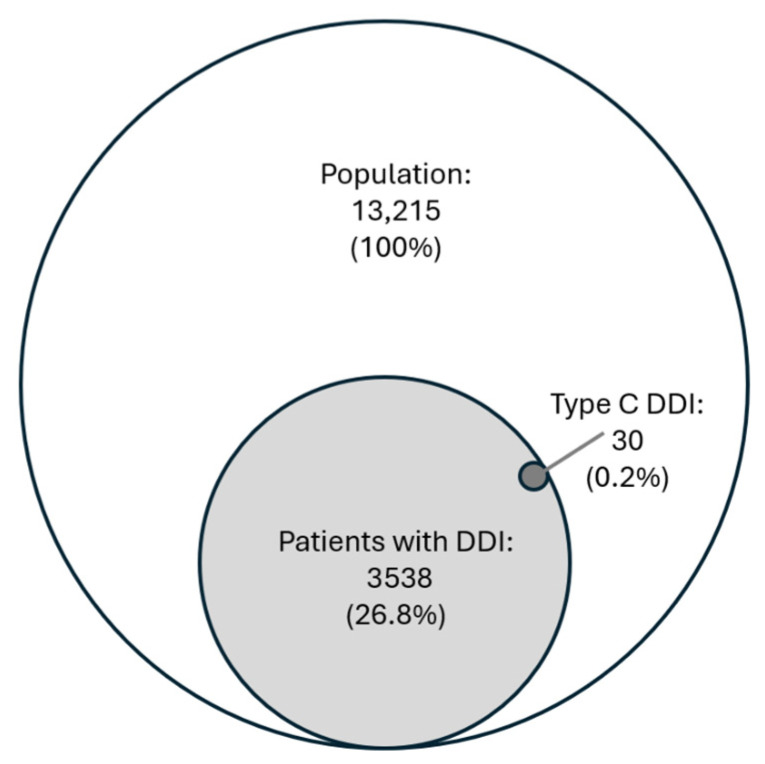
Prevalence of drug–drug interactions (DDIs) with DOAC in the study population. Type C = “should be avoided”.

Figure 2 shows that 26.8% of the study population had at least one medication that interacted with the DOAC. The most common DDIs were with escitalopram (933 patients, 7.0%), acetylsalicylic acid (919 patients, 7.0%) and amiodarone (339 patients, 2.6%).

Table 3 lists all 30 Type C DDIs («Should be avoided»), and the seven most frequently used medications involved in Type B DDIs («Precautions should be taken»). For three of the 30 type C cases the medication list contained a free-text note stating that the two medications should not be taken at the same time. The remaining 27 patients had no such note.

### 3.2. Fall-Risk Increasing Drugs (FRIDs)

In total, 12,785 (96.7%) of patients had at least one FRID or drug that may cause or worsen orthostatism (OD). As shown in Table 4, beta-blocking agents were the most commonly prescribed FRID/OD (69.0%), followed by diuretics (49.7%), renin-angiotensin system inhibitors (43.1%), and hypnotics and sedatives (37.8%).

### 3.3. Anticholinergic Drugs

Table 5 gives an overview of the 5 most commonly prescribed ACs in the study population. According to CALS, 6188 (46.8%) had a total anticholinergic score of ≥3, and the highest individual score observed was 15 (one patient). Among patients with a total score ≥ 3, the median score was 3 and mean score was 4.5. The most commonly used high-scoring drugs in our study were sedating antihistamines (hydroxyzine), tricyclic antidepressants (amitriptyline) and antimuscarinics for overactive bladder (solifenacin).

## 4. Discussion

This study investigates co-prescribing of DOACs and FRIDS, ACs and DDIs in MDD users. Of the 13,215 DOAC users in this study, 26.8% had at least one medication that potentially interacts with their prescribed DOAC, and 0.2% were a Type C interaction (“should be avoided”). Additionally, nearly all patients (97%) were prescribed a FRID medication and about half of the patients had an anticholinergic score of 3 or above.

Our study shows that about 20% of the MDD users in Norway were prescribed DOACs. This is considerably higher than the 6.2% for the equivalent age group in the general population [33] and reflects a greater prevalence of anticoagulant treatment among MDD users. While DOACs in the general population are most frequently used in the age group 75–79 [33,34], they were most commonly used in the age group 85–89 in our study. Apixaban was the most commonly prescribed DOAC in both populations [33,34]. There is still limited research on the relative safety of the different DOACs, especially among the older patients. However, the current evidence suggests that in AF patients, DOACs are associated with a lower risk of stroke, systemic embolism, major bleeding and intracranial bleeding, while the risk of GI bleeding is increased compared to warfarin [35,36]. Among the DOACs, apixaban appears to have the lowest risk of GI bleeding, approximately the same as for warfarin [37,38].

### 4.1. Drug–Drug Interactions

Because we have only looked at DDIs involving DOACs and not the total number of DDIs in the population, it is difficult to compare our results to other studies. However, previous studies on DDIs in MDD users generally reveal many DDIs, but few serious DDIs in this population [39,40], which is similar to what we have found in our present study.

In total, 3538 (26.8%) of the study population had at least one medicine that had a DDI with the DOAC they were prescribed. Only 30 (0.2%) of the identified DDIs were classified as severe and recommended to be avoided, including the combination of a DOAC with warfarin. However, because our study is cross-sectional and parts of the medication list include prescribed but not necessarily dispensed medicines, the simultaneous presence of both agents on a patient’s medication card does not prove concurrent administration, but rather may reflect a transition period during switching between anticoagulants. Indeed, in three of the 30 Type C cases both agents were present on the list at the same time, but a free-text note explicitly warned that the two medicines should not be taken at the same time, illustrating that some apparent contraindications are managed in practice by timing or monitoring. The remaining 99% of the DDIs were classified as “precautions should be taken”. As shown in Table 3, combining any of the DOACs with acetylsalicylic acid (7.0%) or amiodarone (2.6%) is among the most common DDIs in our population. These combinations illustrate situations where clinical appropriateness of the combinations can differ depending on the patient. For example, patients with coexisting coronary artery disease (CAD) and atrial fibrillation (AF) may need co-administration with DOAC and antiplatelet therapy (simple or double), which gives an elevated risk of bleeding, and at the same time lowers the high ischemic risk [41,42]. Wu et al. [43] found that the combination of DOAC and amiodarone, but not dronedarone (both antiarrhythmic drugs), increases the risk of major bleeding, in particular intracranial bleeding. However, concomitant prescribing of antiarrhythmic drugs and DOAC is indicated for some patients with atrial fibrillation. The guidelines recommend avoidance or dose reduction in edoxaban (DOAC) when co-prescribed with dronedarone [44]. In this case, the concomitant use may be clinically justified because the risk of unwanted bleeding is compensated for by reducing the dose of edoxaban but not stopped. Jobsky et al. found that in a nursing home population, there is a need for monitoring the risk of bleeding when DOACs are co-prescribed with, for instance, antibiotics or antiplatelet medications [45].

It is not possible, based on our data, to assess whether the necessary precautions have been taken to adjust dosage or if a risk–benefit assessment has been made. Another challenge in regard to dosage of DOAC and DDI is that the correlation between DOAC serum concentrations and effect is not evident [46]. It is also difficult to assess the overall bleeding risk for patients with multiple DDIs. This is even more complicated for older patients because they have a slower elimination of drugs and are more likely to experience adverse effects [42,45,47].

### 4.2. Falls

In our study, nearly all (96.7%) of the 13,215 patients used drugs that increase the risk of falling or worsen orthostatism (FRIDs). In Table 4, we see that the most common FRIDs are beta-blocking agents (68.4% of patients), diuretics (49.3%) and RAS (renin-angiotensin system) inhibitors (43.0%). This reflects that they are prescribed medication to treat AF and other cardiovascular diseases, which is often indicated in older patients [44,48].

On the negative side of prescribing FRIDS, Meade et al. found that older DOAC users experiencing low-level falls had a higher risk of intracranial bleeding. Additionally, the use of DOACs was connected to increased mortality [49,50]. Contrary to prescribing FRIDs in combination with DOACs, Galvain et al. found [51] that DOAC was associated with fewer intracranial hemorrhages and ischemic strokes and systemic embolism than warfarin. If anticoagulation is required, DOAC is a safer option. Bell et al. found that GPs paid little attention to the risk of prescribing FRIDs to older adults, unless the patients reported dizziness or that they had fallen [52]. Also, to reduce serious adverse effects such as intracranial bleeding during falls, regular medication reviews are crucial to prevent harm.

### 4.3. Anticholinergic Drugs

Some of the adverse effects of anticholinergic (AC) drugs are dizziness, impaired cognitive function, constipation, dry mouth, urine retention and palpitations [47]. Of the 13,215 patients in our study, 6188 (46.8%) had an AC score of three or more. Table 5 shows the agents contributing most to the burden. Some of these medications such as digoxin and essential cardiovascular therapies are necessary and with clear indications for use. However, we see that several of the higher-scoring agents such as hydroxyzine, quetiapine and amitriptyline contribute disproportionately to high individual totals but are also potentially modifiable by deprescribing or substitution. These classes thus represent logical targets in medication reviews. Consistent with our findings, a recent Norwegian registry study found that anticholinergic and sedative drug burden was significantly associated with post-discharge institutionalization in older, acutely admitted community-dwelling patients; the analysis indicated higher sensitivity for anticholinergic (AC) drugs and suggested that reducing the number of AC agents may lessen the risk of institutionalization [53]. This supports the clinical relevance of our anticholinergic burden measure and reinforces that identifying and prioritizing higher-impact AC agents (those contributing most to individual scores) should be an explicit component of medication review and deprescribing efforts.

The prevalence of anticholinergic drug use in older patients varies greatly between studies (range 10–80%); however, the prevalence is lower for drugs with a high anticholinergic score [22,54,55]. Our study population had an average age of 84, and the use of ACs in older adults has been shown to impair cognitive performance and increase mortality risk [56,57]. Previous studies have shown that patients with a high anticholinergic burden have an increased risk of falling [22,23,24]. As discussed above, an increased risk of falls may raise the likelihood of intracranial hemorrhages, which can be more serious in patients using DOACs.

Considering the high proportion of FRIDs and anticholinergic drug use in our study, including increased risks of anticholinergic adverse effects, the GPs should be encouraged to assess the anticholinergic burden for each patient by for instance using tools such as Beers Criteria [58] and the STOPP-START Criteria [59] to optimize the patients’ medicine list and to reduce the risk of unwanted anticholinergic effects which induce falls, and, in the worst case, cause intracranial bleedings or fractures.

### 4.4. Implications

In 2020, the Norwegian Prescription Database reported that 4.8 times more DOACs were dispensed than warfarin. Over the years, not only have DOACs replaced warfarin, but the overall prevalence of patients receiving anticoagulant treatment has also increased. From 2010 to 2020, the combined use of warfarin and DOACs rose from 18 to 32 per 1000 inhabitants [33]. While this increase may be positive, as previous research has indicated under-treatment [60,61], there are concerns about potential over-treatment [61,62]. Given our findings of frequent co-prescribing of FRIDs and ACs alongside DOACs, particularly in the oldest patients, clinicians should consider individual risk–benefit assessments. While some co-prescription in our study are considered necessary for individual patients, reflecting established cardiovascular indications, our findings also highlight that a high proportion of patients are prescribed multiple drugs that increase the risk of falls and/or serious bleeding events. This emphasizes the importance of an overall medication review and assesses the overall risk, rather than focusing solely on individual medicines. Our findings support a holistic geriatric assessment that combines systematic medication review (including deprescribing where appropriate), targeted nutritional evaluation and routine use of simple laboratory markers to detect malnutrition and better prioritize modifiable contributors to pharmacologic risk. Table 3, Table 4 and Table 5 show medications classified as both FRID and AD, along with potential DDIs with DOACs. By evaluating the necessity of these medications and focusing on modifiable contributors such as for example sedative-hypnotics or urological agents where there are safer alternatives without DDIs, small changes in a patient’s medication regimen can greatly impact the risk of bleeding and side effects in older adults. Given the high age of our study population, it is also crucial to consider the risk–benefit ratio of medication use in general and to consider deprescribing [63].

Tools to identify FRIDs and ACs, such as STOPPFall [59], CRIDECO Anticholinergic Load Scale (CALS) [31], ADS and ACB scales [32], are available as tools; however, they are not integrated into the software that physicians use during prescribing. Also, some prescribers have limited knowledge of such tools [52]. An exception is the Norwegian Medicines Agency tool for DDIs, which is integrated in the software and routinely used by GPs and pharmacists, which may explain the low prevalence of serious DDIs found in our study. Many patients are also unaware that some medications they take increase the risk of falling [64]. To reduce the prescribing medications that increase the risk of harms, increased knowledge and awareness of such side effects for healthcare personnel and patients are needed. We recommend that tools for identifying such problems be integrated into daily practice and prescribing support software.

### 4.5. Strengths and Limitations

A major strength of this study is that it includes almost 90% of all MDD users in Norway, providing a comprehensive overview of anticoagulation use in this population. Additionally, the inclusion of patients in nursing homes, a group that is not included in the Norwegian Prescription Database [33], adds value to our study.

However, some limitations regarding using data from the MDD prescriptions are important to be aware of. For medications dispensed as MDD (tablets and capsules), the prescription card used in this study represents dispensing records, meaning that these medications were dispensed at the same time. However, other medications listed on the card represent prescribing records. There might be medications listed that the patient is no longer using, and vice versa—the patient may use medications they have at home, but for which there is no longer a valid prescription. This can lead to both over- and underestimations of DDIs, FRIDs, and ACs in our study. Importantly, this study is based on cross-sectional MDD medication lists and is not linked to clinical outcome registries or patient medical records. Thus, while we identify potential DDIs, FRIDs and elevated anticholinergic burden, we cannot determine whether these led to adverse events (e.g., falls, bleeding or hospitalization) or whether co-prescribing was clinically justified. These limitations mean our results should be interpreted primarily as a description of medication patterns and potential pharmacological risks rather than evidence of causation or clinical outcome.

## 5. Conclusions

The findings provide real-world description of DOAC co-prescribing in MDD users and may help limit the use of medication combinations that increase pharmacologic risk—specifically anticholinergic drugs, fall-risk increasing drugs and drugs that have drug–drug interactions with DOAC. The results have also revealed that several of the very oldest patients use DOACs. The benefits of DOAC treatment must be addressed at an individual level, with attention to symptoms such as any bleeding in skin or soft tissue.

## Figures and Tables

**Table 1:** Study population characteristics and drug use (*n* = 13,215).

	DOAC	Apixaban	Rivaroxaban	Dabigatran	Edoxaban
Users (percent)	13,215 (100%)	9083 (68.7%)	3041 (23.0%)	1041 (7.9%)	50 (0.4%)
Mean number of drugs * (min–max)	10.3 (1–32)	10.3 (1–32)	9.9 (1–32)	10.7 (2–27)	9.6 (1–21)
Mean age (min–max)	85.0 (65–107)	85.4 (65–104)	84.2 (65–107)	84.2 (65–103)	84.9 (65–99)
Sex					
Male	5083 (38.5%)	3427 (67.4%)	1227 (24.1%)	406 (8.0%)	23 (0.5%)
Female	8132 (61.5%)	5656 (69.6%)	1814 (22.3%)	635 (7.8%)	27 (0.3%)
Care setting					
Home care services	9977 (75.5%)	6774 (67.9%)	2253 (22.6%)	906 (9.1%)	44 (0.4%)
Nursing home	2614 (19.8%)	1876 (71.8%	651 (24.9%)	83 (3.2%)	4 (0.2%)
Self-pay home-dwelling	624 (4.7%)	433 (69.4%)	137 (22.0%)	52 (8.3%)	2 (0.3%)

* Unique items on the prescription card, including dietary supplements, prnmedications, medical devices and consumables (e.g., diabetes supplies; incontinence products).

**Table 2:** The use of FRIDs, anticholinergic medicines, and DDIs in home care services, nursing homes and home dwelling patients (*n* = 13,215).

	DDI	FRID	AC	No of Drugs (Mean)	Female (%)	Age (Mean)
Home care services (*n* = 9977)	2703 (27.1%)	9735 (97.6%)	4856 (48.7%)	10.9	61.4	84.8
Nursing home (*n* = 2614)	609 (23.3%)	2436 (93.2%)	1059 (40.5%)	7.7	65.1	86.8
Self-pay home-dwelling (*n* = 624)	226 (36.2%)	614 (98.4%)	273 (43.8%)	10.2	49.0	81.4
Total (*n* = 13,215)	3538 (26.8%)	12,785 (96.7%)	6188 (46.8%)	10.3	61.5	85.0

**Table 3:** The 7 most frequently used medications involved in drug–drug interactions (DDI) with DOAC.

Type C DDI: Should Be Avoided (*n* = 30)		Type B DDI: Precautions Should Be Taken (*n* = 4073)	
Medication Name (ATC Code)	Number of Patients	Medication Name (ATC Code)	Number of Patients
Warfarin (B01AA03)	10	Escitalopram (N06AB10)	933
Enoxsaparin (B01AB05)	7	Aspirin (B01AC06)	919
Heparin (B01AB01)	4	Amiodarone (C01BD01)	339
Dalteparin (B01AB04)	2	Verapamil (C08DA01)	288
Carbamazepin (N03AF01)	3	Sertraline (N06AB06)	245
Phenytoin (N03AB02)	2	Venlafaxsine (N06AX16)	216
Phenobarbital (N03AA02)	2	Clopidogrel (B01AC04)	212

**Table 4:** Frequency and percentage of ATC groups for fall risk inducing drugs (FRIDs) and drugs that may cause of worsen orthostatism (ODs). *n* = 13,215.

ATC Code—Therapeutic Drug Group	Study Population (*n* = 13,215)		Male (*n* = 5083)		Female (*n* = 8132)	
Fall-risk inducing drugs	*n*	(%)	*n*	(%)	*n*	(%)
N05C—Hypnotics and sedatives	4997	(37.8)	1628	(30.3)	3369	(40.6)
N02A—Opioids	3655	(27.7)	1137	(21.2)	2518	(30.4)
N06A—Antidepressants	3053	(23.1)	995	(18.5)	2058	(24.8)
N05B—Anxiolytics	2858	(21.6)	832	(15.5)	2026	(24.4)
N05A—Antipsychotics (lithium excluded)	1001	(7.6)	363	(6.8)	638	(7.7)
Drugs that may cause or worsen orthostatism						
C07—Beta blocking agents	9124	(69.0)	3462	(64.4)	5662	(68.2)
C03—Diuretics	6571	(49.7)	2395	(44.6)	4176	(50.3)
C09—Renin-angiotensin system inhibitors	5698	(43.1)	2272	(42.3)	3426	(41.3)
C08—Calcium channel blockers	2502	(18.9)	915	(17.0)	1587	(19.1)
C01D—Vasodilators used in cardiac diseases	2330	(17.6)	889	(16.5)	1441	(17.4)
G04CA—Alpha-adrenoreceptor antagonists	900	(6.8)	900	(16.7)		(0.0)
N04B—Dopaminergic agents	418	(3.2)	200	(3.7)	218	(2.6)
C02—Antihypertensives	139	(1.1)	90	(1.7)	49	(0.6)

**Table 5:** The 5 most commonly prescribed anticholinergic drugs with an anticholinergic score of 1, 2 and 3 respectively. (*n* = 13,215).

Score 1	Users	Score 2	Users	Score 3	Users
Metoprolol (C07AB02)	7267	Tramadol (N02AX02)	1125	Hydroxyzine (N05BB01)	203
Furosemide (C03CA01)	2882	Quetiapine (N05AH04)	382	Amitriptyline (N06AA09)	190
Oxazepam (N05BA04)	2110	Ranitidine (A02BA02)	220	Solifenacin (G04BD08)	157
Digoxin (C01AA05)	1241	Olanzapine (N05AH03)	141	Tolterodine (G04BD07)	93
Prednisolone (H02AB06)	1187	Baclofen (M03BX01)	72	Levomepromazine (N05AA02)	69

## Data Availability

The data that support the findings of this study are provided by Apotek 1 Gruppen AS and are not publicly available. Data are however available from the corresponding authors upon reasonable request and with permission of Apotek 1 Gruppen AS.

## Abbreviations

The following abbreviations are used in this manuscript:

DOAC	Direct oral anticoagulant
MDD	Multidose drug dispensing
DDI	Drug–drug interaction
FRID	Fall-risk increasing drug
AC	Anticholinergic drug
